# Effect of radiotherapy sequence on long-term outcome in patients with node-positive breast cancer: a retrospective study

**DOI:** 10.1038/s41598-022-14873-9

**Published:** 2022-06-24

**Authors:** Joohyun Woo, Byung-In Moon, Hyungju Kwon, Woosung Lim

**Affiliations:** Department of Surgery, Ewha Womans University College of Medicine, Ewha Womans University Mokdong Hospital, 1071 Anyangcheon-ro, Yangcheon-gu, Seoul, Korea

**Keywords:** Cancer, Breast cancer, Cancer therapy

## Abstract

The optimal sequence of chemotherapy (CT) and radiotherapy (RT) after surgery in breast cancer patients is unclear. There is a lack of literature on RT given between anthracycline and taxane administration. We evaluated the effect of RT sequence on long-term outcome in breast cancer. Two hundred patients who underwent surgery between January 2009 and December 2012 for node-positive breast cancers were evaluated retrospectively. All patients were treated with doxorubicin and cyclophosphamide (AC) followed by taxane. Sandwich RT group that received RT between AC and taxane was compared to the group that received RT after CT. The mean follow-up period was 105.4 months. The locoregional recurrence (LRR) rate was lower in sandwich RT group (P = 0.012) and there was no significant difference in distant metastasis between the two groups. The RT sequence was an important predictor for LRR in multivariable analysis (P = 0.017). For luminal A subtype, disease-free survival (DFS) was better in sandwich RT group than in CT followed by RT group (P = 0.001). The overall survival did not correlated with RT sequence regardless of subtype. Sandwich RT can offer DFS benefit in luminal A subtype breast cancer. A tailored approach of sequencing chemotherapy and radiotherapy would be needed considering the factors that can influence outcome.

## Introduction

Radiotherapy (RT) reduces 5-year locoregional recurrence rates by 17–19% and 15-year breast cancer mortality rates by 5.4% in early breast cancer. Recent studies demonstrated that RT made significant differences in breast cancer-specific survival at 15 years and those are approximately proportional to the differences in 5-year local recurrence rates^1^. RT can improve local control and survival by eradicating local tumor deposits that may persist after surgical removal^2^.

Individualized RT planning and delivery is important according to the 3.2021 version of the NCCN guidelines for breast cancers. Also CT is used as a systemic therapy in patients with node-positive HER2-positive or triple-negative breast cancer and with N2/N3 disease of hormone receptor (HR)-positive and HER2-negative breast cancer. CT can directly impact distant metastasis and mortality but RT can affect long-term mortality by the decrease in local recurrence rates. Therefore, depending on disease state or surgical method, the optimal sequence of chemotherapy and radiotherapy after surgery for breast cancer patients requiring both RT and CT is not clear although RT is commonly recommended to follow chemotherapy (CT) when CT is indicated^3^.

There are several randomized trials and reviews on the sequence of CT and RT, which are concurrent chemoradiation or sequential^4–6^. However, the information these studies provide is limited as the patients included in these studies were treated in the early 2000s and the results do not reflect the current treatment modalities. RT between chemotherapy courses, which is sandwich RT, interrupts CT. This treatment schedule has different effects on disease outcome according to the chemotherapy regimen employed^7–10^. Research on sandwich RT is limited to cyclophosphamide, methotrexate, and fluorouracil (CMF) or anthracycline regimens and strong evidence of the effect of sandwich RT in patients treated with AC followed by taxane, which is the standard therapy for node-positive breast cancers, is not yet provided.

We conducted a retrospective study to investigate the effect of RT sequence after surgery on cancer recurrence and patient survival in node-positive breast cancer cases. In this paper, we report long-term outcome of sandwich RT compared to conventional RT.

## Methods

### Patient selection

A total of 894 patients underwent breast-conserving surgery (BCS) or mastectomy for invasive breast carcinoma by three breast surgeons between January 2009 and December 2012. Sentinel lymph node biopsy was performed for axillary staging in all patients. Of the 388 patients with lymph node-positive breast cancer, 263 patients who received axillary lymph node dissection and were treated with adjuvant doxorubicin and cyclophosphamide (AC) followed by taxane (T) chemotherapy and radiotherapy (RT) were selected for this study. The medical records were reviewed to identify patients and their sequence of CT and RT. The difference in chemotherapy and radiotherapy sequence was dependent on physician. Patients with unknown treatment start date or with micrometastases in lymph nodes were excluded. Patients who did not complete CT and RT as planned were also excluded. We analyzed retrospectively 200 patients, which were divided into a group receiving RT after CT (CT followed by RT group) and a group receiving RT between AC and T chemotherapy (sandwich RT group) according to sequence of CT and RT.

The collected patient clinical data were age; tumor size and number of positive lymph nodes; histological grade and presence of lymphovascular invasion; EIC; estrogen receptor (ER), progesterone receptor (PR), and human epidermal growth factor receptor 2 (HER2) status; and ki-67 index. ER, PR, and HER2 status in tumors were determined using IHC according to the FDA-approved Allred scoring system for HER2. HER2 positivity was defined as either 3 + on IHC staining or positive fluorescence in situ hybridization signal. The molecular subtype was classified as follows: ER + or PR + , HER2−, and low ki-67 (< 20%) (luminal A); ER + or PR + , and HER2 + or high ki-67 (≥ 20%) (luminal B); HR- and HER2 + (HER2 enriched); or ER-, PR-, and HER2- (triple negative).

### Treatment

Adjuvant CT was started within three weeks following BCS or mastectomy. All patients received surgical treatment by three breast surgeons in a single hospital and CT was administered with a same protocol according to department policies. RT was started after 4 cycles of AC or after 4 cycles of taxane following 4 cycles of AC, which was determined according to their clinicians’ practices. In the CT followed by RT group, patients were treated with four cycles of CT consisting of doxorubicin 60 mg/m^2^ combined with cyclophosphamide 600 mg/m^2^ every three weeks followed by four cycles of paclitaxel 175 mg/m^2^ or docetaxel 75 mg/m^2^ every three weeks. RT was started within four weeks after the last CT drug administration. In sandwich RT group, RT began four weeks after the 4th cycle of AC administration, and the 1st cycle of paclitaxel or docetaxel was administered in three weeks after completion of RT. Patients who received BCS underwent whole-breast irradiation and tumor-bed boost and supraclavicular lymph-node radiation therapy. If the internal mammary lymph nodes were suspicious for metastasis, they were included in the radiation field. The dose of irradiation was 50.4 Gy for the whole breast, 50.4 Gy for supraclavicular nodes in 28 fractions, and 10 Gy for tumor bed in 5 fractions. Patients who received mastectomy underwent radiotherapy of 50.4 Gy for chest wall, supraclavicular nodes, and intermammary nodes in 28 fractions.

### Stastistical analysis

The SPSS software version 20 (SPSS, Chicago, IL, USA) was used for all statistical analyses. Basic data on the characteristics of study subject groups were compared and analyzed using Chi-squared tests. The mean values of the groups were compared with each other using independent sample t-tests (Student t-test) to examine statistical significance. Tumor recurrence in the ipsilateral breast or chest wall of patients treated or in ipsilateral axillary, subclavicular, supraclavicular lymph nodes, or internal mammary nodes was classified as locoregional recurrences. Any recurrence at distant sites including contralateral axillary, subclavicular, supraclavicular, or internal mammary lymph nodes was regarded as distant metastasis. The association between clinicopathologic parameters and locoregional recurrence rate within each group was evaluated using the Chi-squared test and univariable Cox regression analysis. Furthermore, multivariable Cox regression analysis was used to determine the independent prognostic factors within the stratified cohorts. Disease free survival (DFS) was defined as the time from surgery to the detection of the first locoregional recurrence or distant metastasis. Overall survival (OS) was defined as the time from surgery to death from any cause. Survival curves were assessed using the Kaplan–Meier method and comparison of survival curves was analyzed using the log-rank test. Multivariable analyses were conducted by using Cox’s proportional hazard regression model. Factors associated with them with a P-value of less than 0.20 in the univariable analysis were entered in the model for multivariable analysis. When the correlation between some factors was very high (correlation coefficient > 0.8–0.9) by the multicollinearity test, only one was included in the model for multivariable analysis. A P value of < 0.05 for a two-sided test was considered statistically significant, whereas a P value of 0.05 to 0.10 was described as borderline significant. This study was approved by the institutional review board of the Ewha Clinical Trial Center at Ewha Womans University Medical Center, and written informed consent was obtained from all the patients.

### Ethical approval

All procedures performed in studies involving human participants were in accordance with the ethical standards of the institutional review board of the Ewha Clinical Trial Center at Ewha Womans University Medical Center and with the 1964 Helsinki declaration and its later amendments or comparable ethical standards. Informed consent was obtained from all individual participants included in the study.

## Results

### Clinicopathological characteristics

A total of 200 patients were analyzed in this study. The CT followed by RT group included 90 patients and sandwich RT group has 110 patients. As shown in Table 1, there was no significant difference in clinicopathologic parameters in both groups. More than two-thirds of patients in each group underwent breast-conserving surgery. Axillary lymph node dissection was performed in more than 90% of participants in both groups. Tumor size were less than 5 cm in more than 90% of patients in each group. Negative margins were obtained in all patients. N3 disease was found in 14.4% of patients in CT followed by RT group and 10.9% of patients in sandwich RT groups. The luminal A type was the most common (47.7% vs. 39.1%) and HER2 enriched type was the least (2.3% vs. 9.1%), but there was no significant difference between two groups. There was a borderline significant difference in drug used for taxane chemotherapy: docetaxel was used in 22.2% of patients in CT followed RT group and in 34.5% of patients in sandwich RT group (P = 0.062).

**Table 1:** Clinicopathological parameters.

	CT followed by RT	Sandwich RT	P-value
	(N = 90)	(N = 110)	
Age (years)	47.9 ± 9.0 (27–73)	49.2 ± 9.2 (30–72)	0.306
**Breast surgery**			0.757
Conserving	71 (78.9)	75 (68.2)	
Mastectomy	19 (21.1)	35 (31.8)	
**Axillary surgery**			0.757
SLNB	4 (4.4)	7 (6.4)	
ALND	86 (95.5)	103 (93.6)	
**Histology**			0.807
Ductal	76 (84.4)	95 (86.4)	
Lobular	6 (6.7)	5 (4.5)	
Other	8 (8.9)	10 (9.176)	
**ER**			0.256
Positive	64 (71.1)	86 (78.2)	
Negative	26 (28.9)	24 (21.8)	
**PR**			0.410
Positive	71 (78.9)	81 (73.6)	
Negative	19 (21.1)	29 (26.4)	
**HER2**			0.375
Positive	15 (16.7)	25 (22.7)	
Negative	75 (83.3)	85 (77.3)	
**T stage**			0.260
T1	31 (34.4)	51 (46.4)	
T2	51 (56.7)	49 (44.5)	
T3	8 (8.9)	9 (8.2)	
T4	0 (0.0)	1 (0.9)	
**N stage**			0.774
N1	51 (56.7)	65 (59.1)	
N2	22 (24.4)	30 (27.3)	
N3	13 (14.4)	12 (10.9)	
**TNM stage**			0.851
II	52 (57.8)	65 (59.1)	
III	38 (42.2)	45 (40.9)	
**Histologic grade**			0.501
Grade I	18 (20.0)	17 (15.5)	
Grade II	41 (45.6)	48 (43.6)	
Grade III	31 (34.4)	45 (40.9)	
**EIC**			0.497
Yes	18 (20.0)	27 (24.8)	
No	72 (80.0)	82 (75.2)	
**Lymphovascular invasion**			0.488
Yes	57 (63.3)	67 (60.9)	
No	32 (35.6)	43 (39.1)	
Unknown	1 (1.1)	0 (0.0)	
**Ki-67**			0.147
< 20%	45 (53.3)	58 (52.7)	
≥ 20%	39 (43.3)	52 (47.3)	
Unknown	3 (3.3)	0 (0.0)	
**Subtype**			0.197
Luminal A	42 (47.7)	43 (39.1)	
Luminal B	29 (33.0)	48 (43.6)	
HER2	2 (2.3)	10 (9.1)	
TNBC	15 (17.0)	9 (8.2)	
**Taxane**			0.062
Paclitaxel	70 (77.8)	72 (65.5)	
Docetaxel	20 (22.2)	38 (34.5)	

Data are presented as No. (%) unless otherwise specified.

*SLNB* sentinel lymph node biopsy, *ALND* axillary lymph node dissection, *ER* estrogen receptor, *PR* progesterone receptor, *HER2* human epidermal growth factor receptor 2, EIC Extensive intraductal component, *TNBC* triple negative breast cancer.

### Locoregional recurrence and distant metastasis

Mean interval between surgery and start of radiotherapy was 5.5 ± 1.2 months in CT followed by RT group and 3.0 ± 0.8 months in sandwich RT group. In patients who received endocrine therapy, mean of interval between surgery and start of endocrine therapy was 5.6 ± 0.9 months in sandwich RT group and 7.6 ± 1.1 months in CT followed RT group. The mean follow-up period of all patients was 105.4 months (range 7–160). Four patiens (3.6%) displayed locoregional recurrence in sandwich RT group, which was significantly lower than 12 patients (13.3%) in CT followed RT group. Eight patients (8.9%) in CT followed RT group and 3 patients (2.7%) in sandwich RT group developed recurrence to ipsilateral breast or chest wall. Ipsilateral breast or chest wall relapse occurred more frequently than ipsilateral axillary and regional nodal relapse in both groups. There was one patient with relapse on both ipsilateral chest wall and axillary and regional nodes in each group. Twenty two patients (24.7%) in CT followe RT group and 19 patients (17.3%) in sandwich RT group developed distant metastasis. There was no significant difference between two groups. Locoregional recurrence as the first recurrence was more common in CT followed RT group and distant metastasis as the first recurrence was more common in sandwich RT group although it was borderline significant (P = 0.067) (Table 2). In both univariable and multivariable analysis, RT sequence was found to be a significant predictor for locoregional recurrence (Table 3).

**Table 2:** Recurrence and death.

	CT followed by RT	Sandwich RT	P-value
	(N = 90)	(N = 110)	
Mean follow-up period (months)	105.4 (7–160)		
**Loco-regional recurrence**	12 (13.3)	4 (3.6)	0.012
Ipsilateral breast/chest wall	8 (8.9)	3 (2.7)	
Ipsilateral axillary and regional nodes	5 (5.6)	2 (1.8)	
Distant metastasis	22 (24.7)	19 (17.3)	0.220
**First recurrence**			0.067
Loco-regional recurrence	9 (36.0)	4 (18.2)	
Distant metastasis	16 (64.0)	18 (81.8)	

Data are presented as No. (%) unless otherwise specified.

**Table 3:** Univariable and multivariable analysis of loco-regional recurrence.

Variables	Univariable		Multivariable	
	OR (95% CI)	P-value	OR (95% CI)	P-value
Age < 50 vs. ≥ 50	0.388 (0.121–1.249)	0.112	3.027 (0.905–10.132)	0.072
BCS vs. Mastectomy	1.662 (0.455–6.074)	0.443	NI	
ER-negative vs. -positive	1.404 (0.463–4.257)	0.549	NI	
PR-negative vs. -positive	1.490 (0.491–4.527)	0.481	NI	
HR-negative vs. -positive	2.243 (0.728–6.913)	0.159	2.568 (0.781–8.443)	0.120
HER2-positivie vs. -negative	0.549 (0.120–2.520)	0.440	NI	
Tumor size > 5 cm vs. ≤ 5 cm			NI	
N3 vs. N1-2	1.626 (0.429–6.162)	0.475	N	
TNM stage III vs. II	1.891 (0.946–3.781)	0.071	0.593 (0.191–1.843)	0.367
Histologic grade III vs. I-II	1.285 (0.458–3.607)	0.634	NI	
Ki-67 > 20% vs. ≤ 20%	1.275 (0.640–2.540)	0.490	NI	
Paclitaxel vs. Docetaxel	1.246 (0.385–4.036)	0.714	NI	
AC-RTx-T vs. AC-T-RTx	0.245 (0.076–0.789))	0.018*	0.236 (0.072–0.774)	0.017*
Subtype (vs..Luminal A)		0.461	NI	
Luminal B	0.914 (0.268–3.125)	0.886		
HER2	1.197 (0.131–10.900)	0.873		
TNBC	2.633 (0.678–10-229)	0.162		

*BCS* breast-conserving surgery, *SLNB* sentinel lymph node biopsy, *ALND* axillary lymph node dissection, *ER* estrogen receptor, *PR* progesterone receptor, *HR* hormone receptor, *HER2* human epidermal growth factor receptor 2, *EIC* Extensive intraductal component, *TNBC* triple negative breast cancer, *NI* not included.

### Disease-free survival and overall survival

In univariable analysis, negative ER, negative PR, N3 disease, and molecular subtype were prognostic factors for DFS. Although only of borderline significance (P = 0.051), RT sequence was also a prognostic factor for DFS. In multivariable analysis, luminal A subtype and RT between AC and T chemotherapy was related to better DFS (Table 4). There were 34 deaths (17.0%); 19 patients (21.6%) died in the CT followed by RT group and 15 patients (13.8%) died in sandwich RT group. Negative ER, negative PR, histologic grade III, and TNBC subtype significantly correlated with poor OS by univariable analysis. When these variables were assessed by multivariable analysis, molecular subtype was identified as the only factor that correlated with OS (P = 0.008) (Table 5). RT sequence was not correlated with OS in univariable and multivariable analysis.

**Table 4:** Univariable and multivariable survival analysis of disease free survival.

Variables	Univariable		Multivariable	
	HR (95% CI)	P-value	HR (95% CI)	P-value
Age < 50 vs. ≥ 50	1.018 (0.548–1.892)	0.954	1.039 (0.547–1.971)	0.908
BCS vs. mastectomy	1.227 (0.622–2.417)	0.555	NI	
ER-negative vs. -positive	2.405 (1.264–4.576)	0.007*	NI	
PR-negative vs. -positive	2.370 (1.242–4.523)	0.009*	NI	
HER2-positivie vs. -negative	1.480 (0.702–3.121)	0.303	NI	
Tumor size > 5 cm vs. ≤ 5 cm	1.225 (0.479–3.135)	0.671	NI	
N3 vs. N1-2	2.446 (1.193–5.015)	0.015*	1.835 (0.838–4.021)	0.129
TNM stage III vs. II	1.316 (0.717–2.415)	0.376	NI	
Histologic grade II vs. I-II	1.809 (0.970–3.374)	0.062	0.985 (0.439–2.214)	0.971
Ki-67 > 20% vs. ≤ 20%	1.667 (0.891–3.119))	0.110	NI	
Paclitaxel vs. Docetaxel	1.382 (0.741–2.579)	0.309	NI	
AC-RTx-T vs. AC-T-RTx	0.535 (0.286–1.003)	0.051	0.461 (0.236–0.902)	0.024*
Subtype (vs..Luminal A)		0.012*		0.144
Luminal B	1.389 (0.650–2.971)	0.396	1.494 (0.633–3.524)	0.359
HER2	3.080 (0.998–9.510)	0.050	3.298 (0.849–12.812)	0.085
TNBC	3.743 (1.579–8.872)	0.003*	3.018 (1.076–8.467)	0.036*

*BCS* breast-conserving surgery, *SLNB* sentinel lymph node biopsy, *ALND* axillary lymph node dissection, *ER* estrogen receptor, *PR* progesterone receptor, *HER2* human epidermal growth factor receptor 2, *EIC* Extensive intraductal component, *TNBC* triple negative breast cancer, *NI* not included.

**Table 5:** Univariable and multivariable survival analysis of overall survival.

Variables	Univariable		Multivariable	
	HR (95% CI)	P-value	HR (95% CI)	P-value
Age < 50 vs. ≥ 50	0.780 (0.372–1.636)	0.511	0.894 (0.416–1.919)	0.773
BCS vs. Mastectomy	1.017 (0.448–2.310)	0.968	NI	
ER-negative vs. -positive	3.962 (1.884–8.333)	0.000*	NI	
PR-negative vs. -positive	1.228 (2.010–8.892)	0.000*	NI	
HER2-positivie vs. -negative	1.246 (0.505–3.076)	0.633	NI	
Tumor size > 5 cm vs. ≤ 5 cm	1.225 (0.479–3.135)	0.671	NI	
N3 vs. N1-2	1.918 (0.778–4.730)	0.157	NI	
TNM stage III vs. I-II	1.976 (0.935–4.179)	0.075	1.573 (0.691–3.576)	0.280
Histologic grade II vs. I-II	2.715 (1.271–5.798)	0.010*	1.336 (0.499–3.574)	0.564
Ki-67 > 20% vs. ≤ 20%	1.892 (0.886–4.042(	0.100	NI	
Paclitaxel vs. Docetaxel	0.537 (0.254–1.135)	0.103	0.726 (0.312–1.691)	0.458
AC-RTx-T vs. AC-T-RTx	0.723 (0.344–1.518)	0.391	0.642 (0.285–1.450)	0.287
Subtype (vs..Luminal A)		0.000*		0.010*
Luminal B	1.177 (0.413–3.358)	0.760	1.010 (0.311–3.281)	0.987
HER2	5.141 (1.503–17.589)	0.009*	3.759 (0.803–17.595)	0.093
TNBC	6.100 (2.318–16-047)	0.000*	4.543 (1.386–14.887)	0.012*

*BCS* breast-conserving surgery, *SLNB* sentinel lymph node biopsy, *ALND* axillary lymph node dissection, *ER* estrogen receptor, *PR* progesterone receptor, *HER2* human epidermal growth factor receptor 2, *EIC* Extensive intraductal component, *TNBC* triple negative breast cancer, *NI* not included.

**Figure 1:**
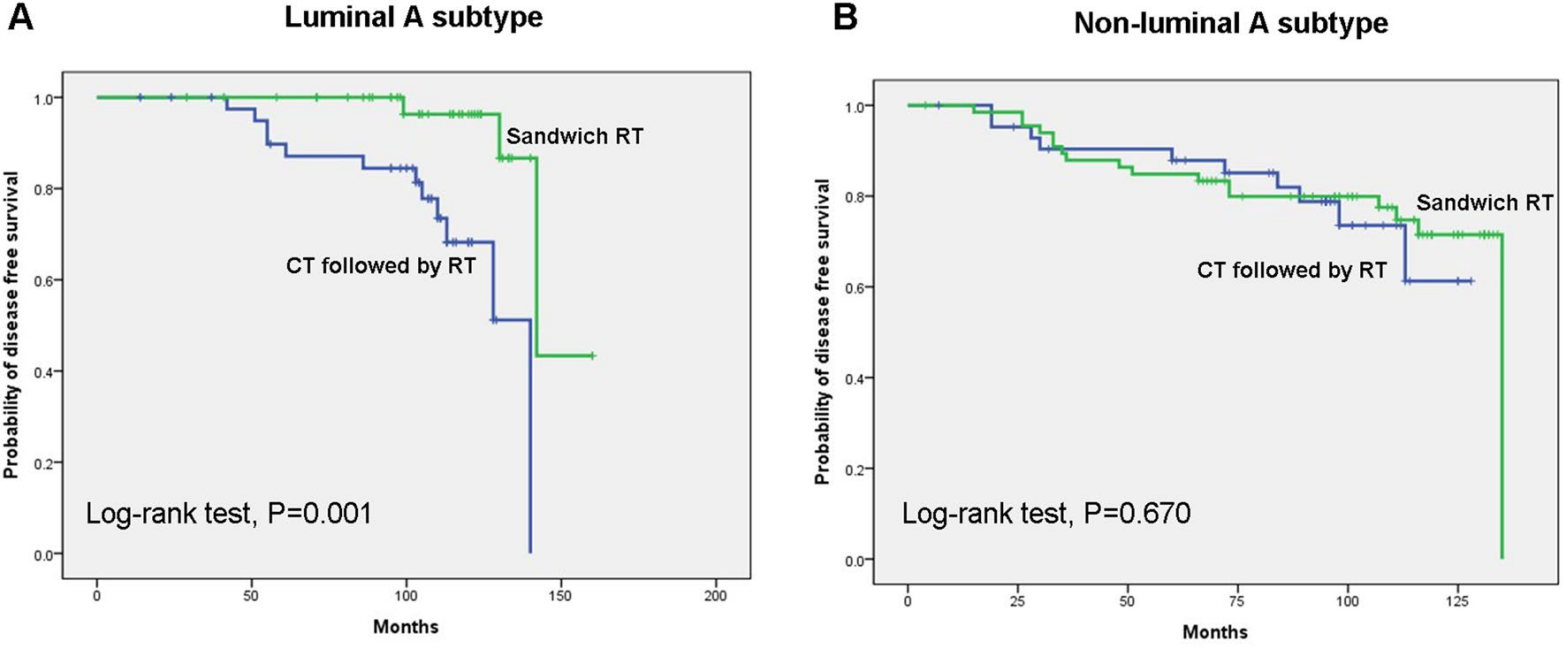
Disease free survival according to sequence of RT in luminal A subtype (**A**) or non-luminal A subtypes (**B**).

**Figure 2:**
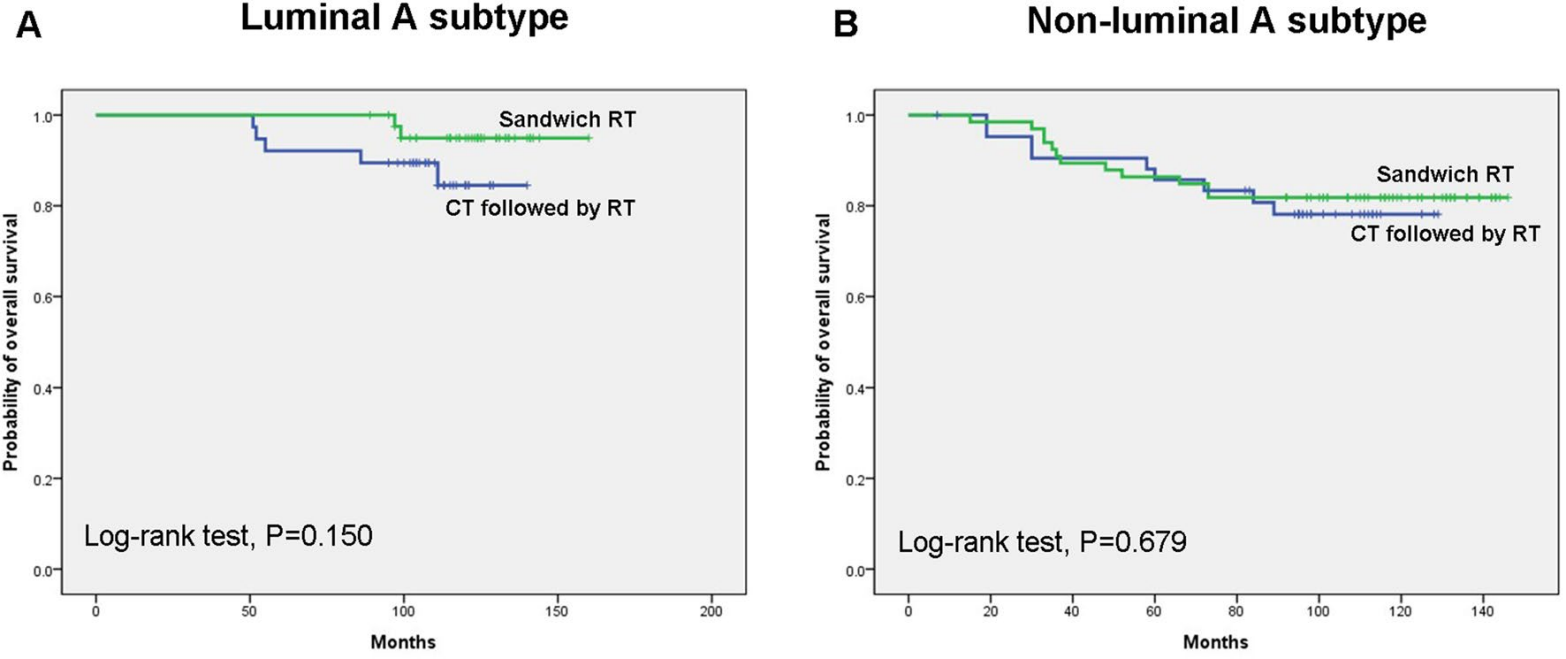
Overall survival according to sequence of RT in luminal A subtype (**A**) or non-luminal A subtypes (**B**).

Subgroup analysis stratified by luminal A or non-luminal A subtype showed that there was a significant relationship between RT sequence and DFS in luminal A subtype, but not in non-luminal subtype (P = 0.001 vs. P = 0.670). For luminal A subtype, DFS was better in sandwich RT group than in the CT followed by RT group (Fig. 1). Regardless of luminal A or non-luminal A subtype, OS was not correlated with RT sequence (Fig. 2).

## Discussion

Our results indicate that sandwich RT contributed to improvement in 8-year DFS including locoregional recurrence, but not in distant metastasis, in node-positive breast cancer patients. The impact on LRR depends on the start of RT rather than of CT. A randomized control trial on sequencing of CT and RT after BCS for patients with four or more positive nodes found that there was a higher local recurrence rate in the CT-first group and a higher distant recurrence rate in the RT-first group. It was analyzed that a distant recurrence rate was high in the RT-first group because the median dose of CT delivered was lower in the CT-first group^11^. The result of effect on LRR is consistent with our study. Also, in our study that analyzed patients who received the same doses of CT, there was no significant difference between the CT followed by RT group and sandwich RT group in distant metastasis or OS. On the other hand, a research suggested that a treatment schedule with CT first cannot compromise local control despite of the delay in RT^12^. A retrospective study showed that delay in starting RT for patients who underwent BCS and received chemotherapy before RT did not compromise 10-year outcomes. However, in this study, patients who received chemotherapy followed by RT and subsequent chemotherapy were not included. All of patients were treated with RT after completion of chemotherapy although time interval between surgery and RT was shorter than 112 days. Patients with N0 disease also were included and axillary lymph node dissection was not performed for some patients with node-positive disease^13^.

RT has impacts on LRR and OS in breast cancer. Several studies suggested that postoperative RT after systemic therapy could improve 15-year breast cancer mortality and OS in node-positive breast cancer patients^1,9,14–16^. Locoregional RT within six months of beginning chemotherapy improved both disease recurrence and mortality in a meta-analysis that reviewed 18 randomized controlled trials with 6,367 participants^17^. In contrast, several randomized clinical trials demonstrated that postoperative adjuvant RT reduced locoregional recurrence but did not affect OS^18,19^. After 10 years of follow-up in MA.20^20^ and in EORTC 22,922^21^, regional nodal irradiation was found associated with improvement in locoregional and disease-free survival and lower breast cancer mortality, but not overall survival. RT sequence did not affect 8-year OS in our study. The reason why RT sequence did not correlate with OS, even though high-risk patients were analyzed in this study, may be that all of the patients received RT and there was a relatively small difference of interval from surgery to RT between the two groups. The eight years of follow-up and the small number of patients studied may also be the cause.

The timing of effective RT varies. The interval from surgery to the start of RT is determined according to the CT and RT sequence. In our study, RT was started 5.5 months after surgery in the CT followed by RT group and there was an impact on LRR despite of the difference of 2.5 months between the two (assessed) groups. Hickey et al. reported that starting RT within 8 months after surgery did not reduce survival^16^. Huang et al. demonstrated that patients who received RT for more than 8 weeks after breast cancer surgery were 2.28 times more likely to develop locoregional recurrences^22^. A systemic review suggested that the delay in RT caused an increase in local recurrence but it was not related to distant metastasis or OS regardless of whether patients received CT or not^23^. The present study also showed that the timing of RT could affect the local recurrence. Moreover, it had an impact on DFS and OS. Delayed RT, 6 months after surgery, increased local recurrence rate and led to significantly poorer OS and DFS^24^.

It is important to note whether chemotherapeutic drugs are administered while RT is delayed. As CT has a crucial effect on survival as a systemic therapy and RT has an established role in the prevention of local recurrence, which treatment begins first can influence disease outcome in patients who have risk factors for recurrence. The results from randomized trials of CT followed by RT versus RT followed by CT showed that there is no significant difference between RT sequences in mortality and local or distant recurrence^16^. A recent meta-analysis showed that survival and recurrence in patients who received sandwich RT was not significantly different from that of patients who received concurrent RT. In contrast, the OS of patients who underwent concurrent chemoradiation was superior to that of sequential RT. It was found that recurrence was higher when chemotherapy was started before radiotherapy than when radiotherapy was initiated first. Avoiding delay in RT or no maintaining no gap between chemotherapy and RT might lead to their additive interaction and tumor response^25^. Our study found that sandwich RT decreased LRR and improved DFS despite all of the cases having negative margins and node-positive disease. These results may support the theory that sandwich method with less delay in starting RT can improve survival and reduce recurrence.

The evidence for effectiveness of sandwich RT is limited^11,24,26,27^. Most previous studies on sandwich RT have included patients undergoing chemotherapy with CMF or anthracycline regimen. RT was delivered between CT cycles in these studies^7,8^. These may support that a sandwich RT can be applied with the CMF regimen but RT insertion between cycles of chemotherapy using anthracycline is not recommended^28^. We showed that sandwich RT between AC and taxane can provide DFS benefit regardless of BCS or mastectomy. Taxanes added sequentially with AC chemotherapy improved DFS despite the delay in starting RT. Henderson et al. suggested that taxanes were the main factors that led to the improvement in survival compared in the only AC chemotherapy followed by RT group and the AC plus taxanes followed by RT group^29^. In our study, we showed the effect of only RT sequence, excluding addition of taxanes on DFS.

We found that sandwich RT improved LRR and DFS, especially in luminal A subtype in the subgroup stratified by molecular subtypes. This result is consistent with the data of Wang et al., which suggested that adjuvant radiotherapy reduced the risk of relapse in luminal A breast cancers^30^. The benefits from chemotherapy were generally smaller in luminal A breast cancers^31^. As the potential effect of CT is relatively small in luminal A subtype, it may not be able to dilute the impact of RT sequence on treatment outcome although while RT is delivered is delivered could allow the proliferation of micr-metastatic disease^22^. The effect of RT on OS in our study was different from the study of Mao et al. who analyzed patients treated with or without RT and showed a significant survival benefit after radiotherapy in younger patients (age at diagnosis < 60 years) with luminal A subtype^32^. Some patients with little benefit from CT might be included in this study because multigenomic assays used to identify patients at increased risk for distant recurrence could not be performed during this study period. However, clinically high-risk tumors with node-positive disease were more frequently high-risk by multigenomic assays^33^.

In the multivariable analysis of our study, RT sequence was a stronger factor for LRR rather than the extent of breast resection and the status of the resection margins which are related to the tumor burden after surgery. The RT sequence of whether sandwich RT or CT followed by RT can affect the length of time required for the remaining tumor cells to proliferate before radiation therapy. These findings can support that delaying RT, while chemotherapy is treated first, could increase local recurrence rates and delaying of systemic chemotherapy. As RT is more effective leading to less residual tumor burden, the residual tumor regrowth after surgery can increase because of a long interval between surgery and RT and lead to poorer outcomes^22^.

This study support that RT can be given between anthracyclines and taxanes in node-positive luminal A subtype breast cancer patients and it can improve locoregional recurrence free survival. Delay of starting endocrine therapy did not affect overall survival when CT and RT were completed. Although literature on effect of RT sequence on toxicity or quality of life is very limited, theoretically, optimal timing of CT and RT can alleviate toxicity. As subsequent CT may increase hematological toxicities of initial chemotherapy, sandwich RT may make these toxicities to be more manageable. It may lead to improve quality of life when subsequent CT is administered and to make it easier to complete full-dose CT without reducing drug dose on the planned schedule. Since more effective local control can be beneficial only in patients at risk for local recurrence among luminal A subtype patients, there is a need to identify more robust predictors of local recurrence such as the expression profiling^34^.

Our study has several limitations. Because of the small sample size, especially for the HER2 subtype, we could not analyze each four subtypes and compare luminal A subtype with non-luminal A subtype, including luminal B, HER2, and TNBC. Given the small numbers of events occurred, it was difficult to determine statistical significance. Also the use of docetaxel was slightly different between the two groups although patients of two groups had similar baseline characteristics and all of patietns were treated with anthracyclin and taxane. We need to follow disease outcomes in patients included in this study with longer follow-up periods to see if there is any change in the overall survival in longer term. Breast cancer is regarded as a systemic disease that spreads by local extension^35^. Whelan et al. suggested that RT may inhibit secondary systemic spread by reducing locoregional recurrence to improve survival when systemic CT is given^17^.

## Conclusions

Our results suggest that in the presence of adjuvant chemotherapy in breast cancer using anthracycline followed by taxane, sandwich RT can improve DFS by reducing locoregional recurrence, especially in luminal A subtype. A tailored approach of sequencing chemotherapy and radiotherapy would be needed considering the factors that can influence outcome.

## Data Availability

The datasets used and/or analyzed during the current study are available from the corresponding author upon reasonable request.
